# Mycofloral profile and the radiation sensitivity (D_10_ values) of solar dried and gamma irradiated *Pleurotus ostreatus* (Jacq.Ex. Fr.) Kummer fruitbodies stored in two different packaging materials

**DOI:** 10.1002/fsn3.545

**Published:** 2017-11-20

**Authors:** Nii Korley Kortei, George Tawia Odamtten, Mary Obodai, Michael Wiafe‐Kwagyan

**Affiliations:** ^1^ Department of Nutrition and Dietetics School of Allied Health Sciences University of Health and Allied Sciences Ho Ghana; ^2^ Department of Plant and Environmental Biology College of Basic and Applied Sciences University of Ghana Legon Ghana; ^3^ Food Microbiology Division Council for Scientific and Industrial Research‐ Food Research Institute Accra Ghana

**Keywords:** fruitbodies, gamma irradiation, Mycoflora, *P. ostreatus*, packaging materials, polypropylene, polythene

## Abstract

The presence of fungi in our foods poses serious health risks as some genera of fungi may produce certain mycotoxins which have carcinogenic, mutagenic, teratogenic, and immunosuppressive effect on humans and animals alike. Fruitbodies of *Pleurotus ostreatus* were solar dried at a moisture content of 12.5 ± 0.2% and stored in polythene and polypropylene packs, gamma irradiated at doses of 0 (control), 1, 2, 3, 4, and 5 kGy at a dose rate of 1.7 kGy/hr from a Cobalt 60 source (SLL, 515, Hungary) and stored at room temperature 28–30°C for a period of 12 months. Mycological analyses were done at intervals of 0, 3, 6, and 12 months. A total of eleven (11) fungi belonging to eight fungal genera were isolated on both Cooke's and DRBC media; *Aspergillus* (*A. niger, A. flavus, A. fumigatus, A. tamarii*), *Rhizopus* (*R. oligosporus*), *Mucor* (*M. racemosus*), *Fusarium* (*F. oxysporum*), *Penicillium* (*Penicillium sp*.), *Trichoderma* (*T. viride*), and *Rhodotorula sp*. were recorded. There was a significant (*p* < .05) reduction in initial mycofloral population by an average of 2.2 log cycles as well as in species numbers with increasing doses of radiation. Radiation sensitivity (D_10_ values) also ranged between 1.68–2.78 kGy. Gamma irradiation treatment is one way which can enhance food safety through the reduction in potential pathogens and has been recommended as part of a comprehensive program to enhance food safety.

## 1 INTRODUCTION

Undoubtedly, oyster mushrooms (*Pleurotus spp*.) are one of the most popular species of mushrooms in Ghana (Apetorgbor, Apetorgbor, & Nutakor, 2005; Kortei et al., 2014) probably due to its easy method of cultivation and delicious nature as it is used as a recipe in many of our local as well as some international cuisines. They are readily available on the shelves of supermarkets, local shops, markets, farm gates and also from street vendors (Kortei et al., 2014). Owing to their highly perishable nature, it is of the essence to prolong their shelf life by dehydrating to reduce the water activity. It has been reported that microorganisms do not grow on food products with water activity aw below 0.6 (Labuza & Altunakar, 2007; Yan, Sousa‐Gallagher, & Oliveria, 2008).

Generally, foods that are not properly handled and stored are prone to microbial contamination resulting in the occurrence of certain harmful microorganisms which pose a health hazard. According to Schardl, Panaccione, and Tudzynski (2006) a plethora of fungal spores exists in the environment and these fungal spores which when dry, float through the air and find suitable conditions where they can start the growth cycle again (FSIS, USDA, 2006) and actively growing molds. Fungal contamination of food may be one of the more insidious but seldom recognized causes of diseases. Fungi produce mycotoxins which are adaptable and potent in causing some severe diseases such as cancer and furthermore, damage vital organs such as the liver, kidney, and brain (IARC, 1993). A variety of fungi (*Fusarium, Claviceps, Penicillium, Trichothecium, Aspergillus, Cephalosporium*, etc.) may contaminate foods and produce illness with symptoms such as vomiting, diarrhea, headaches, chills, dizziness, and blurred vision. Prevention of fungal invasion of commodities is by far the most effective method of avoiding mycotoxin problems and could be achieved by gamma irradiation.

The long standing use of gamma radiation in the preservation of fruits, vegetables, pulses, cereals, and dried products has shown encouraging results (Addo, 2008; IAEA TECDOC, 2006; Kortei, Odamtten, Obodai,Appiah, Adu‐Gyamfi et al., 2015; Kortei, Odamtten, Obodai, Appiah, & Wiafe‐ Kwagyan, 2015; Kortei, 2015; Odamtten, 1986; Odamtten, Appiah, & Langerak, 1986). The recommendation by the joint FAO/IAEA/WHO Expert Committee on the wholesomeness of irradiated foods gave the impetus for the acceptability of food irradiated up to an overall dose of 10 kGy and the adoption by the Codex Alimentarius Commission for irradiated foods in 1983, has contributed to a wide acceptance of food irradiation as a preservation method. Currently, National Public Health Authorities of 26 countries have guaranteed some 140 unconditional and provisional clearance covering many different products treated with gamma radiation for human consumption (IAEA, 2004). Nevertheless, in order to utilize irradiation as a food processing technology, it is very important to study the radiation sensitivity of contaminating microorganisms since this provides a basis for accurate estimation of lethal/killing doses (Thayer, 2000). Sensitivity to irradiation varies among bacterial and fungal species and is affected by the components of foods and temperature during irradiation and subsequent storage (Adu‐Gyamfi, Appiah, & Torgby‐Tetteh, 2012; Kortei, 2015; Neimira, 2007). The D_10_‐value (decimal reduction dose) is the radiation dose required to inactivate 90% of a viable microbial population or reduce the population by a factor of 10 (Smith & Pillai, 2004). Estimation of D_10_‐values may be incorporated into risk assessments for designing processes for reduction in microbial populations in food (Cheroutre‐Vialette & Lebert, 2000).

Yeasts and fungi play a major role in the spoilage of foods in Ghana as their growth on foods can cause major quality problems. Some fungi produce potent mycotoxins which could be carcinogenic, mutagenic, teratogenic, and allergic (Tournas, 2005; Kortei, Odamtten, Obodai,Appiah, Adu‐Gyamfi et al., 2015). A good packaging material should not harbor and support growth of microorganisms and should also be able to maintain the lower water activity of the mushrooms after drying during storage. The objective of this study therefore, was to evaluate the mycofloral profile of solar dried and gamma irradiated *P. ostreatus* fruitbodies stored in two packaging materials and determine their radiation sensitivity (D_10_ values) in vivo.

## 2 MATERIALS AND METHODS

### 2.1 Determination of moisture content

The method prescribed by AOAC, (1995) was employed in the determination of the moisture content.

### 2.2 Enumeration of mycoflora

The decimal serial dilution plate technique was used in estimating fungal populations. About 10 g fresh weight of sample was placed in 250 ml Erlenmeyer flask containing 100 ml sterile distilled water. The mixture was shaken at rev/min in a Gallenkamp Orbital Shaker for 30 min. Aliquot (1 ml) of the suspension was placed in sterile universal bottles (MaCartney tubes) containing 9 ml of 0.1% peptone, and was serially diluted up to 1:10^−3^. The fungal population was enumerated on modified Cooke's medium (Cooke, 1954) and Dichloran Rose Bengal Chloramphenicol (DRBC) agar incubated at 30–32°C for 5 to 7 days for species diversity.

### 2.3 Characterization and Identification of fungal isolates

Fungal isolates were examined under stereo‐binocular microscope (Leica 261, Germany) using the needle mounts technique. Their identification was performed according to macro and micromorphological characteristics. All the isolates were identified up to the species using keys and manuals (Barnett & Hunter, 2006; Larone, 1986; Samson, Hoekstra, & Frisvad, 1995). The percentage (%) occurrence of fungi was calculated by the formular according to Sreenivasa, Dass, and Janardhana (2010).

(1) $$\begin{array}{cc} & \mathrm{Percentage}\;(\%)\;\mathrm{occurence}\;\mathrm{of}\;\mathrm{fungal}\;\mathrm{species}=\\ & \;\frac{\text{Number of fungal species isolated}}{\text{Total number of fungi isolated}}\times 100 \end{array}$$

### 2.4 Packaging materials and storage

The mushroom samples (40 g) each were packaged in either transparent polythene (19 cm x 13 cm) and or in transparent polypropylene (18.5 cm x 12.5 cm x 5 cm) pouches and stored at the prescribed temperatures for up to 12 months.

### 2.5 D_10_ values determination

The D_10_ value is the reciprocal of the slope of the exponential part of a survival curve. This value may also be obtained from Equation (1). The data was subjected to regression analysis. The surviving fractions, log_10_ (*N*/*N*_0_) of microorganisms, was calculated and used as relative changes of their actual viable cell counts. The *D*_10_ values were calculated by plotting log_10_ (*N*/*N*_0_) against dose (*D*) according to the equation:

(2) $$D_{10}=\frac{\text{Radiation Dose (D)}}{\text{log}10(N_{0}-N)}$$

where *N*o is the initial viable count; *N* is the viable count after irradiation with dose *D*;* D* is the radiation dose (Kortei et al., 2014; Kortei, Odamtten, Obodai, Appiah, & Wiafe‐ Kwagyan, 2015 Mohan, Pohlman, & Hunt, 2011). The linear correlation coefficient (*r* ^2^) and the regression equations were also calculated.

### 2.6 Statistical analysis

The values obtained for total fungal counts were transformed to logarithm conversions and subjected to analysis of variance (ANOVA) and means separated by Least Significant Difference (LSD) using SPSS version 9 for windows.

## 3 RESULTS

### 3.1 Mycoflora population

**Figure 1:**
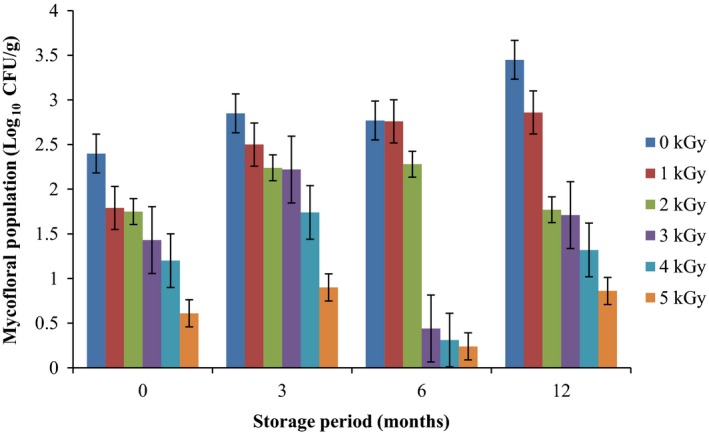
Mycoflora population of mushroom fruitbodies stored in polypropylene packs for up to 12 months and isolated on DRBC medium at 28–32°C

**Figure 2:**
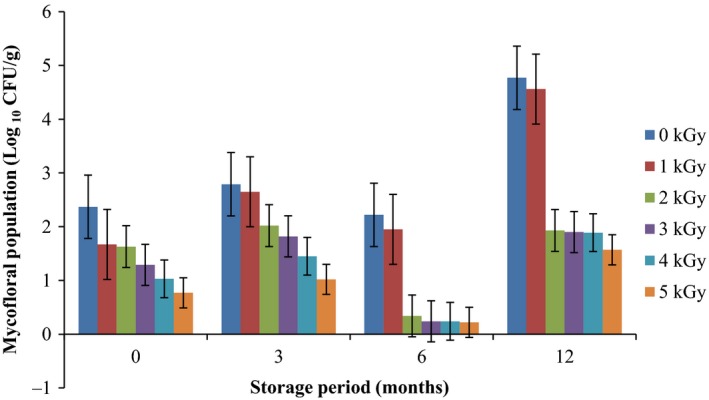
Mycoflora population of mushroom fruitbodies stored in polythene packs for up to 12 months and isolated on DRBC medium at 28–32°C

**Figure 3:**
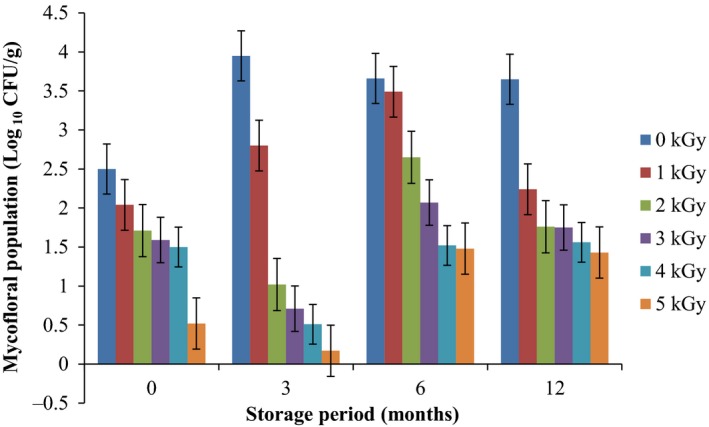
Mycoflora population of mushroom fruitbodies stored in polypropylene packs for up to 12 months and isolated on Cooke's medium at 28–32°C

**Figure 4:**
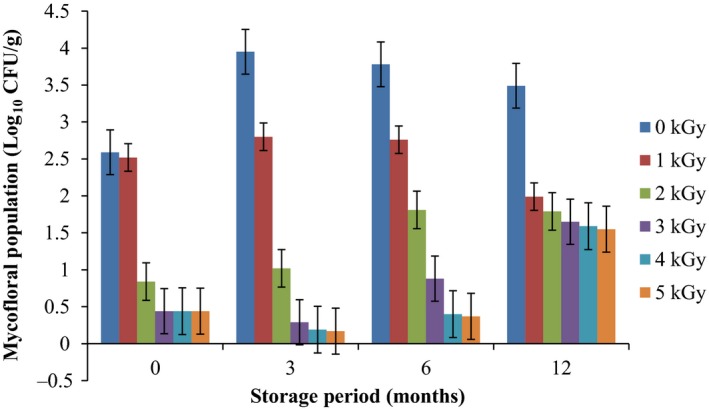
Mycoflora population of mushroom fruitbodies stored in polythene packs for up to 12 months and isolated on Cooke's medium at 28–32°C

Results of the pre and post irradiation mycoflora of dried mushrooms kept in two packaging materials are presented in Figures 1and 2 (on DRBC) and Figures 3and 4 (Cooke's medium). Generally, there was an average of 2.2 log cycles decrease in fungal population of non‐irradiated dry mushrooms by 5 kGy dose in most instances (Figures 1, 2, 3, 4). Response of fungi to radiation in pretreated and non‐pretreated samples differed significantly (*p* < .05).

In the non‐irradiated samples there were significant differences (*p* < .05) between the radiation treatments (1–5 kGy). The same trend was obtained on raising the fungi on Cooke's medium. Although there were increases in fungal population during storage in polythene and polypropylene bags for 3–12 months, the population counts were still low and within the acceptable limits counts (0.86–1.8 log_10_ CFU/g) especially in samples treated with radiation doses 2–5 kGy. Similar trends were recorded for spores incubated on Cooke's medium (Figure 4).

### 3.2 Percentage occurrence of fungal species in the irradiated dried mushrooms

**Figure 5:**
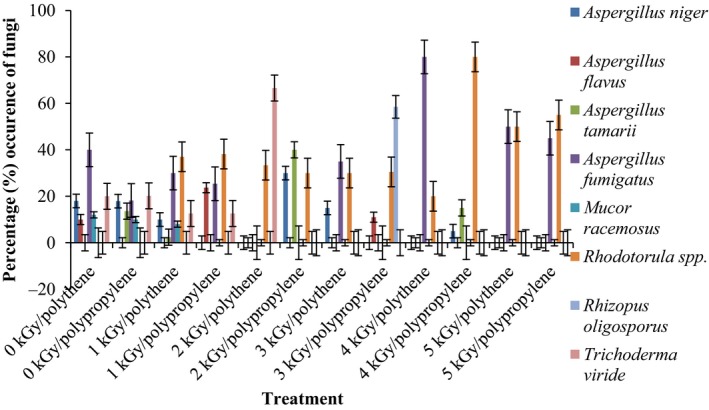
Percentage initial occurrence of fungi on mushroom fruit bodies treated with indicated dosages of gamma irradiation and isolated on Cooke's medium at 28–32°C

**Figure 6:**
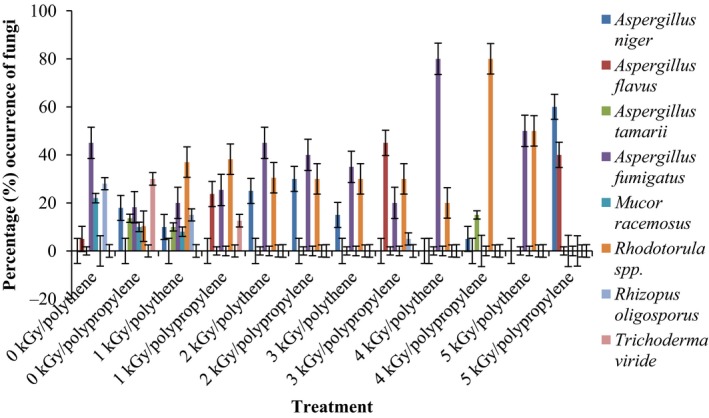
Percentage occurrence of fungi on mushroom fruit bodies stored for 3 months after irradiation in indicated packaging materials and isolated on Cooke's medium at 28–32°C

**Figure 7:**
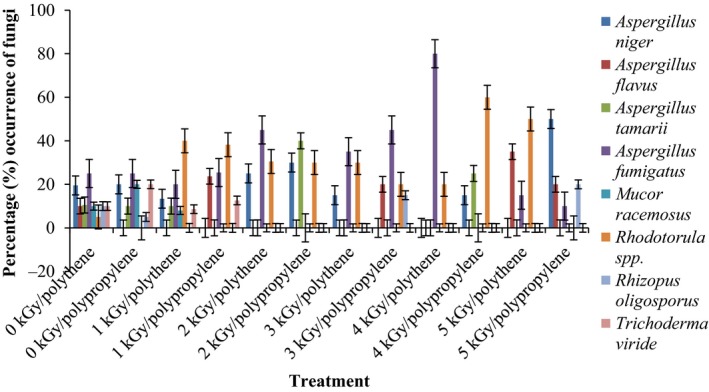
Percentage occurrence of fungi on mushroom fruit bodies stored for 6 months after irradiation in indicated packaging materials and isolated on Cooke's medium at 28–32°C

**Figure 8:**
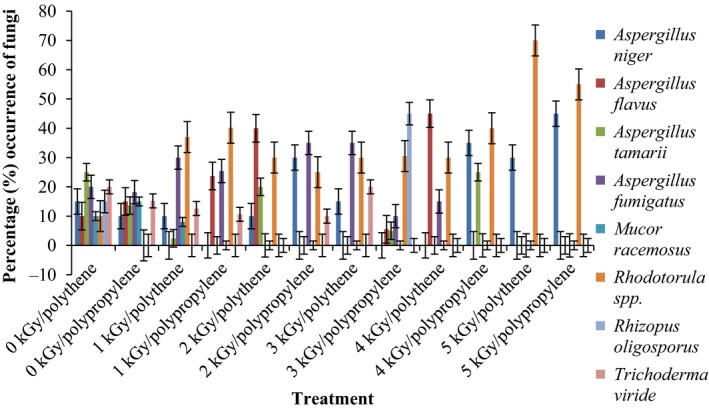
Percentage occurrence of fungi on mushroom fruit bodies stored for 12 months after irradiation in indicated packaging materials and isolated on Cooke's medium at 28–32°C

**Figure 9:**
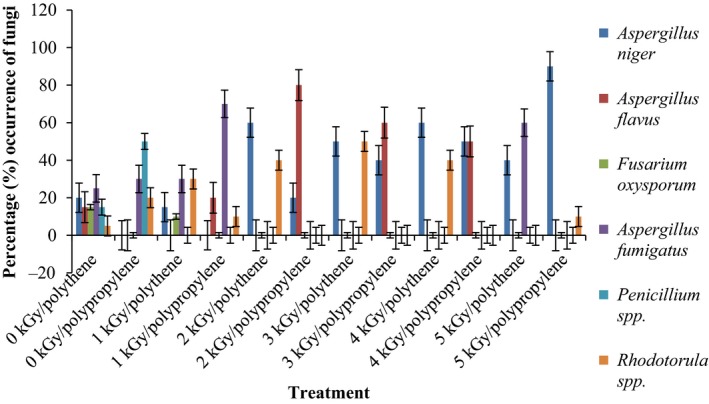
Percentage initial occurrence of fungi on mushroom fruit bodies treated with indicated dosages of gamma irradiation and isolated on DRBC medium at 28–32°C

**Figure 10:**
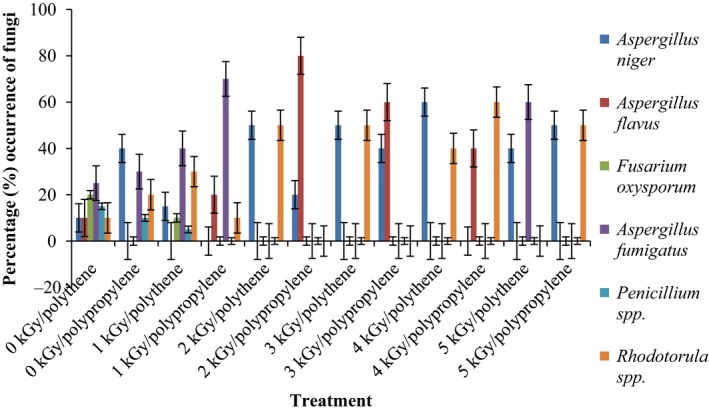
Percentage occurrence of fungi on mushroom fruit bodies stored for 6 months after irradiation in indicated packaging materials and isolated on DRBC medium at 28–32°C

**Figure 11:**
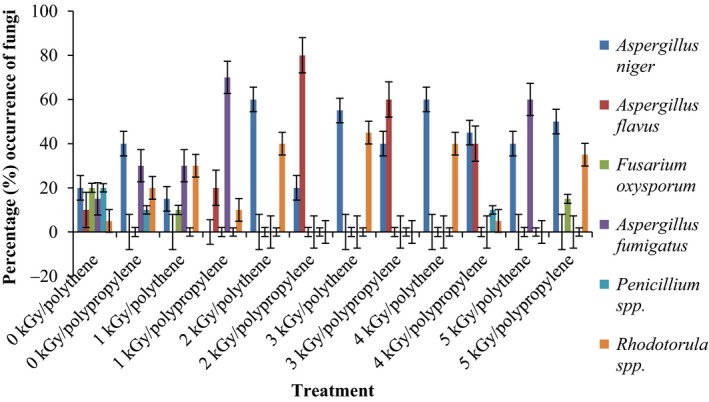
Percentage occurrence of fungi on mushroom fruit bodies stored for 6 months after irradiation in indicated packaging materials and isolated on DRBC medium at 28–32°C

**Figure 12:**
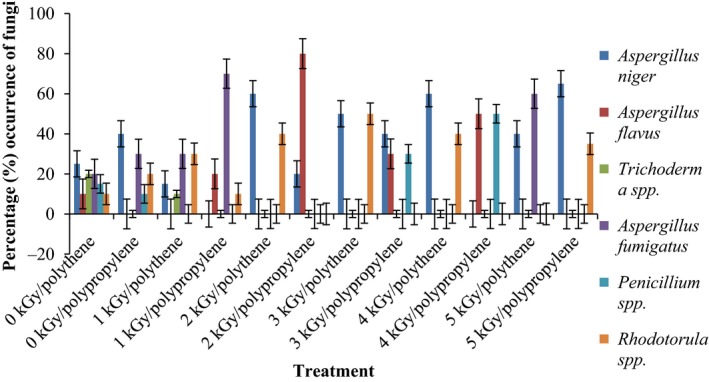
Percentage occurrence of fungi on mushroom fruit bodies stored for 12 months after irradiation in indicated packaging materials and isolated on DRBC medium at 28–32°C

The phenology of the resident fungi in samples stored in polythene and polypropylene packs after radiation (0‐5 kGy) for up to 12 months are presented in Figures 5, 6, 7, 8 (Cooke's medium) and Figures 9, 10, 11, 12 (DRBC). The pooled data of fungi in the two media showed that nine fungal genera *Aspergillus* (*A. niger, A. flavus, A. fumigatus, A. tamarii*), *Rhizopus* (*R. oligosporus*), *Mucor* (*M. racemosus*), *Fusarium* (*F. oxysporum*), *Penicillium* (*Penicillium sp*.), *Trichoderma* (*viride*), and *Rhodotorula sp*. were recorded in the packaging packs after irradiation. *Aspergillus* species predominated over the other species encountered. Notable were two potential toxin‐producing *A. flavus* and *A. fumigatus* and *Penicillium sp*. Species like *A. flavus*,* A. fumigatus*, and *Rhodotorula sp*. persisted on the fruit bodies from an initial >30% but increased to <50% in 12 months.

### 3.3 Radiation sensitivity

**Figure 13:**
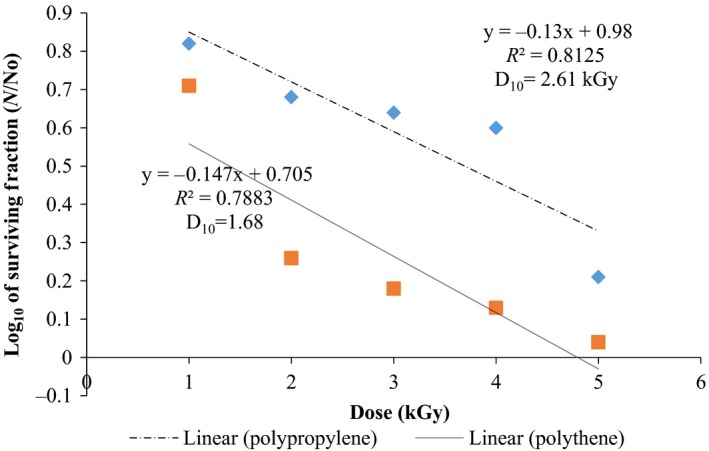
Radiation sensitivity curves of total fungi on mushroom fruit bodies immediately after irradiation and raised on Cooke's medium

**Figure 14:**
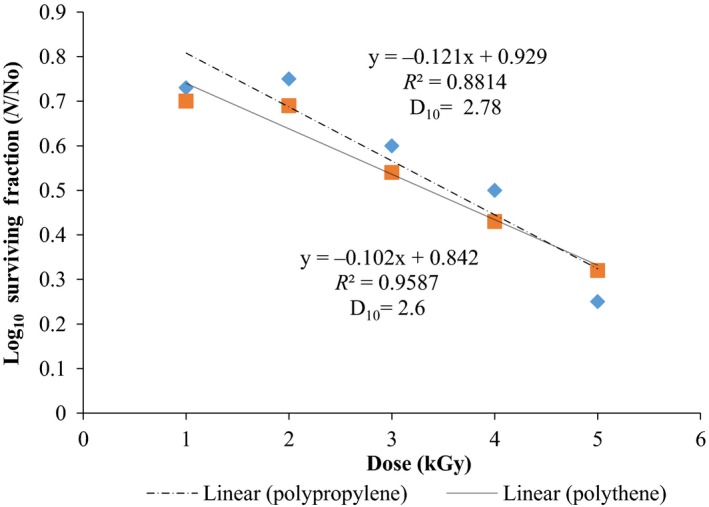
Radiation sensitivity curves of total fungi on mushroom fruit bodies immediately after irradiation and raised on DRBC

Results obtained are presented in Figures 13 and 14. The curves were linear and correlation ratio values *R* ^2^ obtained were positive ranging from *R* ^2 ^= .79–.95 (Table 1). From these curves the D_10_ values were calculated immediately after irradiation with respect to packaging material used. The D_10_ values ranged between 1.68–2.78 kGy depending on the package material. (Figures 13 and 14). The effective dose for killing fungi was close to what was found for *B. cereus* (Kortei et al., 2014) in both packaging packs, that is, D_10 _= 0.76–3.21 kGy (Table 1).

**Table 1:** Mean D_10_ values for fungi resident on mushrooms

Medium	Package	Regression equation (*y*)	*R* ^2^ value	Mean D_10_ value (kGy)
Cooke's	Polypropylene	−0.13*x* + 0.98	.812	2.61^b^
	Polythene	−0.15*x* + 0.705	.788	1.68^a^
DRBC	Polypropylene	−0.121*x* + 0.929	.881	2.78^b^
	Polythene	−0.102*x* + 0.842	.950	2.60^b^

Means with different letters in a column are significantly different (*p* < .05)

## 4 DISCUSSION

### 4.1 Mycoflora population on fruit bodies

Post irradiation storage studies revealed a marginal increase in mycoflora population. Presumably, physical environmental factors such as moisture and temperature in the packs could marginally support growth of microorganisms (Food Safety, 2013).

It was also observed that there was reduction in fungal growth with increasing dose of the irradiated sample, indicating that the radiation could reduce contamination of dehydrated mushroom fruitbodies, no excepting aflatoxigenic and other molds, during the storage time. Presumably, the effect of gamma radiation as sterilizing treatment is to cause direct detrimental effect to cell DNA leading eventually to the killing the spores. McNamara, Black, Beresford, and Parekh (2003) reported that radiation also has an indirect effect as a result of radiolysis of cellular water and formation of active oxygen, free radicals and peroxides causing single and double strand DNA breakages.

Results obtained in this paper agrees favorably with the findings of Kortei et al. (2014), Kortei, Odamtten, Obodai,Appiah, Adu‐Gyamfi, et al., (2015) and Kortei, Odamtten, Obodai, Appiah, & Wiafe‐ Kwagyan, (2015) who recorded 2.4 average log cycles reduction in fungi on dried mushroom fruitbodies and sorghum grains, respectively, using 2.0 kGy of gamma radiation. On the other hand, Ribeiro et al. (2011) showed that a dose of 2.0 kGy was not sufficient to completely reduce the main toxigenic *Aspergillus spp*. resident in soil samples. Although minor changes in the fungal morphology were observed, ultra‐ structural changes at cell wall level and the increase in mycotoxin production ability at 2 kGy were observed.

### 4.2 Percentage (%) occurrence of fungi on fruit bodies

Gamma radiation reduced the percentage (%) occurrence of fungal species. Storage after irradiation showed that non irradiated mushrooms harbored *A. niger*,* A. flavus*,* A. fumigatus*,* M. racemosus*,* Rhodotorula sp*., *T. viride* and *A. niger*,* A. tamarii*,* A. fumigatus*,* M. racemosus*,* R. oligosporus*,* T. viride* in both polythene and polypropylene packs, respectively. Gamma irradiation up to 5 kGy proportionately reduced the incidence of fungi up to 12 months storage although species like *A. flavus, A. fumigatus,* and *Rhodotorula sp*. persisted, (Figures 5, 6, 7, 8, 9, 10, 11, 12) albeit in very low population. The mycotoxigenic potential of *A. flavus* and *A. fumigatus* was not tested but in the case of unlikely availability of favorable environmental conditions they could resume growth and impart mycotoxins like aflatoxin and fumigallin under extremely remote possibilities when the dry fruiting bodies of the mushroom.

Mold fungi isolated in the present work are in agreement with recent works of Anwer, Ali, Hamadamin, and Jaafar (2017) who isolated some molds including *Aspergillus* sp., *Alternaria* sp., *Mucor* sp., *Sachharomyces sp*., *Rhizopus sp*., and *Brettanomyces sp*. from fast food restaurants in Iraq. Annan‐Prah et al. (2011) and Oranusi, Omagbemi, and Eni (2011) isolated similar fungi from street foods and some restaurants in Ghana and Nigeria, respectively.

However, results from Al‐Kahtani (2014) showed a preponderance of *Alternaria* species over *Aspergillus sp*. and *Fusarium sp*. in stored grains in Saudi Arabia. Atanda et al. (2011) suggested that the growth of fungi during storage is controlled to a large extent by; composition of nutrients in the substrate, moisture and temperature conditions and biotic factors like the presence of insects (activities of insects produce moisture) or competition. Majority of the fungal species encountered in this experiment could not survive the radiation treatment. Filamentous fungi range from hypersensitive to extremely resistant with respect to ionizing radiation influence (Mironenko, Irina, Nelli, & Sergey, 2000).

Storage period studies revealed a general significant increase in mycofloral population on the dried mushroom fruit bodies. Kortei (2015) attributed this observation to possibility of certain environmental factors like relative humidity or an increase in water activity becoming conducive to support the growth and survival of the fungi. Results agreed with published findings of Addo (2008) as they investigated the mycofloral profile and aflatoxicogenic potentials of *Aspergillus* species in some packaged Ghanaian foods.

The differences observed between the mycoflora of mushrooms stored in the two packaging materials were not statistically significant (*p* > .05). Nonetheless, for food storage, polypropylene would be the most preferred since it is more robust, aesthetically good and can keep safe from declining quality.

### 4.3 Radiation sensitivity curves of fungi on fruit bodies after irradiation (Cooke's and DRBC)

Radiation sensitivity (D_10_ values) of fungi on dried *P. ostreatus* ranged between 1.68 and 2.61 kGy for samples stored in polythene and polypropylene, respectively, plated on DRBC; D_10_ values of 2.60 and 2.78 kGy were obtained for same samples stored in polythene and polypropylene packs, respectively, and plated on Cooke's medium (Table 1). Statistical differences (*p* < .05) observed could be attributed to the differences in nutrient composition of the growth media and densities of packaging material used for storage.

Results obtained in this work agrees with the report of Frazier and Westhoff (1993) who stated D_10_‐values of 4–11 kGy for yeasts and 1.3–11 kGy for moulds. Recently, Kortei, Odamtten, Obodai, Appiah, & Wiafe‐ Kwagyan, 2015; Kortei, Odamtten, Obodai,Appiah, Adu‐Gyamfi et al., 2015 recorded D_10_ values of 5.64 and 5.94 kGy, respectively, on the influence of gamma radiation and steam sterilizations on the survival of resident fungi of composted sawdust in Ghana. Abouzeid, Abd‐Elrahman, Hassan, Youssef, and Hammad (2003) found that *Aspergillus* and *Penicillium* species were relatively sensitive to ionizing radiation with a D_10_ values between 0.25 and 0.65 kGy; whereas *Fusarium sp*. were more radioresistant requiring high D_10_ values of 0.65 to 1.5 kGy. The problem of mycotoxins imparted into stored food gives cause for the attention of mycotoxins before they are formed by resident fungi. The use of gamma radiation to prevent aflatoxin formation is feasible and may be extended to mushrooms in future studies.

## 5 CONCLUSION

From our studies, it can be surmised that the dried mushroom fruit bodies harbored *Aspergillus sp*. (*A. niger, A. flavus, A. fumigatus, A. tamarii*), *R. oligosporus*,* M. racemosus*,* F. oxysporum*,* Penicillium sp*., *Trichoderma viride*, and *Rhodotorula sp*. There was a significant (*p* < .05) reduction in mycofloral population by > 2 log cycles with the application of gamma irradiation. Gamma irradiation has been proposed as an efficient process to eliminate toxigenic fungi before the initiation of mycotoxins production and has been applied to several foods for extension of shelf life.

The use of irradiation as a complement of the good manufacturing practices (GMP) and may constitute a strategy that could be applied together with other methods to prevent and control the presence of toxicogenic fungi in stored mushrooms.

## CONFLICT OF INTEREST

None declared.
